# Survival Outcomes and Prognostic Factors in Elderly Patients with Hodgkin Lymphoma: A Retrospective Cohort Study

**DOI:** 10.3390/jcm15218305

**Published:** 2026-10-25

**Authors:** Ebru Kavak Yavuz, Yusuf Hekimoğlu, Vehbi Demircan, Abdullah Karakuş, Orhan Ayyildiz

**Affiliations:** 1 Department of Hematology, Medical Faculty, Dicle University, Diyarbakır 21280, Türkiye; 2 Department of Internal Medicine, Kızıltepe State Hospital, Kızıltepe, Mardin 47400, Türkiye

**Keywords:** Hodgkin lymphoma, elderly, comorbidity, International Prognostic Score, neutrophil percentage-to-albumin ratio (NPAR)

## Abstract

**Background:** Hodgkin lymphoma (HL) has a bimodal age distribution, and outcomes in patients aged ≥60 years remain inferior to those in younger cohorts because of a higher comorbidity burden, reduced treatment tolerance, and disease biology that is incompletely characterized in real-world elderly cohorts. **Methods:** We retrospectively analyzed 42 consecutive patients aged ≥60 years with Hodgkin lymphoma. Demographic, clinical, laboratory, and treatment data were extracted from medical records. Overall survival (OS) and progression-free survival (PFS) were estimated using the Kaplan–Meier method and compared with the log-rank test; univariable Cox proportional-hazards regression was used to identify factors associated with OS and PFS. Because complete response and refractory disease are determined only after treatment, and refractory disease is itself a component of the composite PFS endpoint, these two variables were excluded from the Cox and Kaplan–Meier prognostic models and are instead reported descriptively. **Results:** Median age at diagnosis was 68 years (range, 60–94); 71.4% of patients had a high or very-high Charlson Comorbidity Index, and 95.2% presented with B symptoms. Twenty patients (47.6%) died during follow-up; the Kaplan–Meier median overall survival (OS) was 88 months (95% CI, 25–not reached). Progression-free survival (PFS), defined as the composite of relapse, refractory disease, or death from any cause, whichever occurred first, was reached as an event in 21 patients (50.0%); the Kaplan–Meier median PFS was 72 months (95% CI, 8–not reached). On univariable Cox regression, older age (HR 1.09/year, *p* = 0.002), ECOG performance status with >50% of the day spent in bed (HR 22.4 for OS, *p* = 0.003; HR 11.1 for PFS, *p* = 0.002), high International Prognostic Score (IPS) (HR 11.1 for OS, *p* = 0.007; HR 17.7 for PFS, *p* = 0.003), stage IV disease (HR 6.24, *p* = 0.036), splenic involvement (HR 4.33, *p* = 0.002), extranodal involvement (HR 6.55, *p* = 0.001), lower hemoglobin (HR 0.80, *p* = 0.024), lower albumin (HR 0.32, *p* = 0.004), lower Prognostic Nutritional Index (HR 0.93, *p* = 0.006), and higher neutrophil-to-lymphocyte ratio (HR 1.07, *p* = 0.031) were associated with inferior OS. Complete response was achieved in 28 patients (66.7%) and primary refractory disease occurred in 6 patients (14.3%); both showed a marked descriptive association with outcome, but because these variables are assessed after treatment—and refractory disease is itself part of the PFS event definition—they were excluded from the Cox and Kaplan–Meier prognostic models to avoid guarantee-time bias and circularity, and are reported descriptively only. When PFS was defined using this composite endpoint rather than relapse alone, ECOG performance status, IPS risk group, and Ann Arbor stage were each significantly associated with both OS and PFS on Kaplan–Meier/log-rank testing (all *p* < 0.05)—indicating that performance status is not dissociated from progression risk, as a relapse-only PFS definition had previously suggested, but instead reflects both survival endpoints. **Conclusions:** In this real-world elderly HL cohort, performance status, IPS risk group, extranodal/splenic disease involvement, and inflammatory-nutritional biomarkers identified patients at risk of poor survival. The cohort also had a high comorbidity burden (71.4% high-risk and 19.0% very-high-risk Charlson Comorbidity Index), but sparse strata and unstable hazard-ratio estimates meant our data support describing the cohort as highly comorbid without demonstrating a statistically robust CCI–survival association in this dataset. Failure to achieve complete response and primary refractory disease were each associated with markedly worse outcomes descriptively, but were intentionally excluded from the formal prognostic models given their post-treatment, time-dependent nature and circular relationship with the PFS endpoint. These hypothesis-generating findings support incorporating geriatric and biomarker-based risk assessment alongside conventional staging when planning treatment for older patients with Hodgkin lymphoma, and warrant validation in larger prospective cohorts.

## 1. Introduction

Hodgkin lymphoma (HL) is characterized by a bimodal age distribution, with a first incidence peak in young adulthood and a second, less well-studied peak after the sixth decade of life [1,2]. Although the disease is largely curable in young adults treated with doxorubicin, bleomycin, vinblastine, and dacarbazine (ABVD)-based chemotherapy [3] (pediatric patients in Europe are instead treated according to EuroNet-PHL protocols, which are outside the scope of this adult-focused study), older patients continue to experience substantially inferior overall survival, and this age-related outcome gap has persisted despite advances in risk-adapted therapy [4,5].

Several interacting factors contribute to the poor prognosis observed in elderly Hodgkin lymphoma. Older patients more frequently carry a high burden of comorbid disease, as captured by instruments such as the Charlson Comorbidity Index (CCI) [6], which independently predicts both treatment-related mortality and progression-free survival—in some series more strongly than chronological age itself [7]. Reduced bone-marrow and organ reserve limits tolerance of full-dose ABVD, with bleomycin-associated pulmonary toxicity and anthracycline-related cardiotoxicity disproportionately affecting older recipients. This vulnerability stands in contrast to fit younger patients, in whom dose-intensified regimens such as escalated BEACOPP can be used to maximize disease control [8]; in a pooled analysis of two German Hodgkin Study Group (GHSG) trials, omission of bleomycin from ABVD in older early-stage patients was directly shown to reduce pulmonary toxicity without compromising outcome [9]. These observations have motivated growing use of an AVD backbone and of brentuximab vedotin- or PD-1 inhibitor-containing regimens for older or comorbid patients unable to tolerate standard chemotherapy [5]. In addition, unfavorable histologic subtypes (mixed cellularity and lymphocyte-depleted disease) and Epstein–Barr virus-associated tumor biology are more common in older cohorts [10], and functional status assessed by the Eastern Cooperative Oncology Group (ECOG) performance scale or simple frailty indices has repeatedly emerged as one of the strongest single predictors of survival in this age group [11,12].

The International Prognostic Score (IPS), derived from a predominantly younger population with advanced-stage disease, remains the most widely used baseline risk stratification tool in Hodgkin lymphoma [1], but its discriminatory value in patients aged ≥60 years—who differ from the original cohort in comorbidity, treatment intensity, and competing causes of mortality—is less well validated. Recently proposed geriatric-oriented tools, including simplified frailty scores that combine age, ECOG status, and a cumulative illness rating derived from the Cumulative Illness Rating Scale for Geriatrics [13], have shown promising stratification of both treatment-related mortality and survival specifically in older classical Hodgkin lymphoma cohorts [14]. Inflammation- and nutrition-based indices derived from routine blood counts, such as the neutrophil-to-lymphocyte ratio (NLR) [15] and the Prognostic Nutritional Index (PNI) [16], have also been proposed as inexpensive, widely available prognostic markers in Hodgkin lymphoma, reflecting host immune and nutritional status at diagnosis.

Given the persistent challenges in the management of elderly Hodgkin lymphoma and the relative scarcity of granular, real-world data integrating clinical, laboratory, and treatment-response variables in this population, we conducted a retrospective single-center cohort study of patients aged ≥60 years with Hodgkin lymphoma. Our objectives were to (i) describe the demographic, clinical, laboratory, and treatment characteristics of this population; (ii) estimate overall survival (OS) and progression-free survival (PFS); and (iii) identify clinical, laboratory, and treatment-response factors associated with OS and PFS using Kaplan–Meier and univariable Cox proportional-hazards analysis.

## 2. Methods

### 2.1. Study Design and Population

This was a retrospective, single-center cohort study of 42 consecutive patients aged ≥60 years diagnosed with Hodgkin lymphoma. This study was approved by the Ethics Committee of Dicle University on 11 March 2026 (approval no. 222) and was conducted in accordance with the principles of the Declaration of Helsinki; given its retrospective design based on existing medical records, the requirement for individual informed consent was waived by the Ethics Committee. Demographic, clinical, laboratory, treatment, and follow-up data were extracted from institutional medical records. Patients were identified consecutively from institutional records between January 2015 and December 2025, with a data cutoff for follow-up of 28 February 2026. Inclusion criteria were age ≥60 years at diagnosis and a histologically confirmed diagnosis of Hodgkin lymphoma. Patients aged 60 years or younger at diagnosis, those without a definitively confirmed diagnosis of Hodgkin lymphoma, and those with insufficient data for reliable assessment of survival outcomes were excluded from the study. The cohort comprised 41 patients with classical Hodgkin lymphoma (nodular sclerosis, mixed cellularity, lymphocyte-rich, and lymphocyte-depleted subtypes) and 1 patient with nodular lymphocyte-predominant Hodgkin lymphoma (NLPHL); this case is identified separately as “lymphocyte-predominant” in Table 1 rather than grouped with the classical subtypes. The NLPHL diagnosis was confirmed on the basis of histomorphologic findings together with strong CD20 and PAX5 expression and CD15/CD30 negativity in the neoplastic lymphocyte-predominant cells. Because NLPHL is a biologically, immunophenotypically, and clinically distinct entity from classical Hodgkin lymphoma, inclusion of this case introduces a limited degree of histologic heterogeneity into the cohort. In addition, because the International Prognostic Score was developed and validated primarily for advanced-stage classical Hodgkin lymphoma, its prognostic value in the NLPHL case is limited, and the results and risk classification for this case were interpreted with this limitation in mind.

### 2.2. Variables and Definitions

Comorbidity burden was quantified using the Charlson Comorbidity Index [6]. We clarify that the age-weighted scoring component of the Charlson Comorbidity Index was not used in this calculation; age at diagnosis was evaluated as a separate variable in the analyses, so collinearity from including age both within the index and as an independent covariate does not arise. Functional status was recorded using both the Karnofsky Performance Score [17] and the ECOG performance scale [11]. Baseline risk was stratified using the International Prognostic Score (IPS) [1]. Disease stage was assigned according to the Ann Arbor system as adapted by the Lugano classification [18]. Histologic subtype was classified per contemporary WHO criteria into nodular sclerosis, mixed cellularity, lymphocyte-rich, and lymphocyte-depleted subtypes, together with the rarer lymphocyte-predominant variant [19]. Derived hematologic indices were calculated as follows: neutrophil-to-lymphocyte ratio (NLR) [15] = absolute neutrophil count/absolute lymphocyte count; CRP-to-albumin ratio (CAR) = C-reactive protein/albumin; neutrophil percentage-to-albumin ratio (NPAR) [20] = neutrophil percentage/albumin; systemic immune-inflammation index (SII) [21] = platelet count × neutrophil count/lymphocyte count; and Prognostic Nutritional Index (PNI) [16] = (10 × serum albumin [g/dL]) + (0.005 × absolute lymphocyte count [/μL]). Treatment response was categorized as complete response, partial response, or refractory disease; disease refractory to first-line therapy and post-treatment relapse were recorded separately. Response was assessed at interim and/or end-of-treatment PET-CT time points according to the Lugano classification [18]. The event date assigned to primary refractory disease in the time-to-event definitions below was the date of the interim or end-of-treatment PET-CT response assessment at which refractory status was established according to Lugano criteria, since refractory status by definition can only be established after treatment and response assessment, not at diagnosis. Overall survival (OS) was defined as the time from diagnosis to death from any cause or last follow-up. Progression-free survival (PFS) was defined as a composite endpoint: the time from diagnosis to documented post-response relapse, primary refractory disease (dated as above), or death from any cause, whichever occurred first; patients experiencing none of these events were censored at last follow-up. Complete response, partial response, and primary refractory disease are time-dependent variables determined after treatment; to avoid guarantee-time bias, they were not included in Cox regression or Kaplan–Meier analyses starting from the diagnosis date. In addition, because primary refractory disease is a component of the composite PFS endpoint, it was not analyzed as a predictor of PFS. These variables were presented only descriptively (Table 2).

**Table 2:** Treatment, response, and outcome characteristics (*n* = 42).

Characteristic	Value
First-line treatment regimen, *n* (%)	
ABVD	27 (64.3)
AVD	6 (14.3)
BV-AVD	1 (2.4)
R-CHOP	1 (2.4)
GDP	2 (4.8)
No treatment given	2 (4.8)
ABVD × 4 + radiotherapy + DHAP *	1 (2.4)
ABVD × 4 + brentuximab vedotin + nivolumab	1 (2.4)
ABVD × 2 + brentuximab vedotin × 4 + dacarbazine	1 (2.4)
Number of treatment lines, *n* (%)	
1	34 (81.0)
2	4 (9.5)
3	2 (4.8)
4	2 (4.8)
Radiotherapy, *n* (%)	
No	41 (97.6)
Yes	1 (2.4)
Complete response, *n* (%) ‡ (descriptive only—see Section 2.2)	
No	14 (33.3)
Yes	28 (66.7)
Partial response, *n* (%) ‡	
No	40 (95.2)
Yes	2 (4.8)
Refractory disease, *n* (%) ‡ (descriptive only—see Section 2.2)	
No	36 (85.7)
Yes	6 (14.3)
Relapse events, *n* (%)	
None	34 (81.0)
1	5 (11.9)
2	1 (2.4)
3	2 (4.8)
Bleomycin dose reduction, *n* (%)	
None	39 (92.9)
50%	1 (2.4)
100% (discontinued)	2 (4.8)
Doxorubicin dose reduction, *n* (%)	
None	41 (97.6)
75%	1 (2.4)
Pulmonary toxicity, *n* (%) †	
No	34 (94.4)
Yes	2 (5.6)
Cardiac toxicity, *n* (%) †	
No	34 (94.4)
Yes	2 (5.6)
Left ventricular ejection fraction, %, median (range)	60 (30–65)
Observed time to death or last follow-up, months, median (range) §	29.5 (1–159)
Observed time to PFS event or last follow-up, months, median (range) §	21.5 (1–159)
Vital status at last follow-up, *n* (%)	
Alive	22 (52.4)
Deceased	20 (47.6)

**Figure 1:**
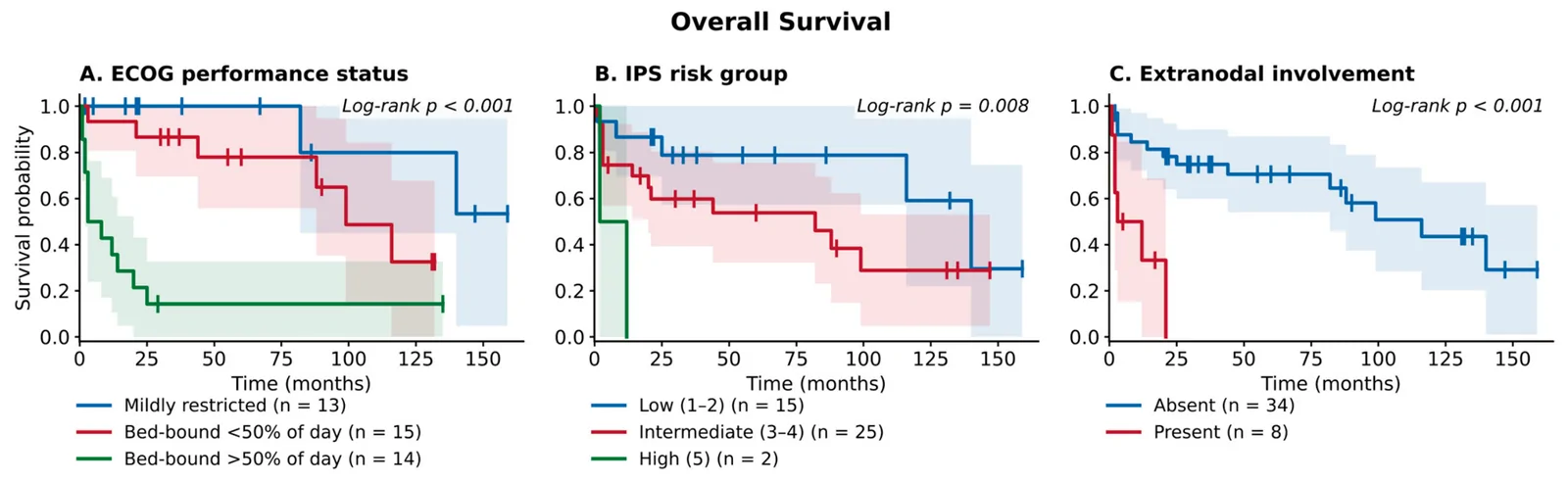
Kaplan–Meier curves for overall survival by (**A**) ECOG performance status, (**B**) IPS risk group, and (**C**) extranodal involvement. Shaded bands represent 95% confidence intervals; tick marks denote censored observations. *p*-values are from the log-rank test. The complete-response panel included in an earlier version of this figure has been removed because complete response was excluded from the prognostic analyses (Section 2.2).

**Figure 2:**
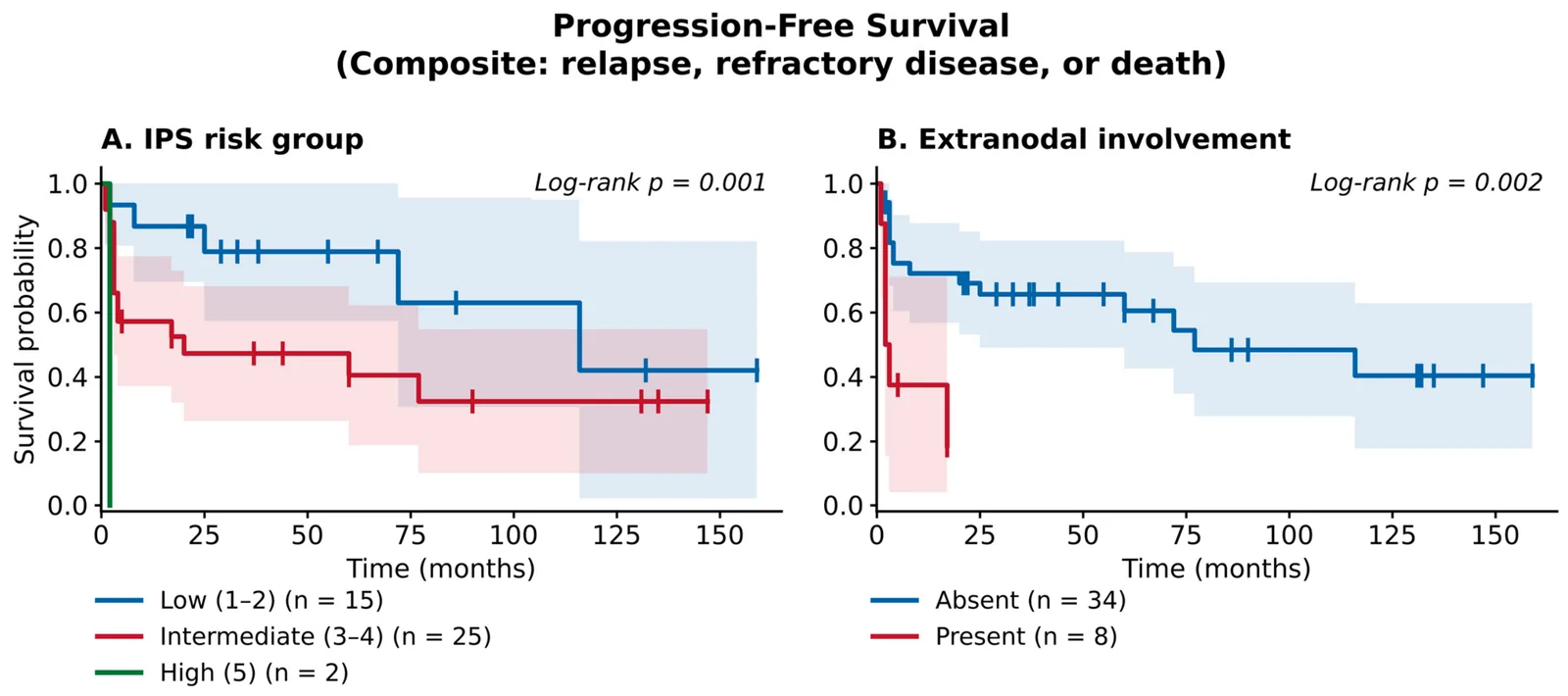
Kaplan–Meier curves for progression-free survival by (**A**) IPS risk group and (**B**) extranodal involvement. Shaded bands represent 95% confidence intervals; tick marks denote censored observations. *p*-values are from the log-rank test. The complete-response and refractory-disease panels included in an earlier version of this figure have been removed because both variables were excluded from the prognostic analyses (Section 2.2).

* DHAP: Dexamethasone, High-dose Cytarabine (Ara-C), and Cisplatin. † Pulmonary and cardiac toxicity were evaluable in 36 of 42 patients; percentages are calculated on the evaluable denominator. ‡ Complete response, partial response, and refractory disease were recorded as separate, non-mutually exclusive binary variables in the source dataset rather than categories of a single response variable; percentages therefore do not sum to 100% across these three characteristics. Complete response and refractory disease are reported here descriptively only and were excluded from the Cox and Kaplan–Meier prognostic analyses in Tables 3, 5 and 6 and Figure 1 and Figure 2 (Section 2.2). § These are raw observed times (from diagnosis to death/event or last follow-up), not Kaplan–Meier survival estimates; the Kaplan–Meier median OS and PFS for the cohort are 88 and 72 months respectively (Abstract; Section 3.3, Section 3.4 and Section 3.5).

### 2.3. Statistical Analysis

Statistical analysis, data management, visualization, and reporting were performed in R (version 4.5.2). Reusable object-oriented analysis workflows were implemented with the R6 package; hypothesis testing was performed with rstatix; and publication-ready summary and analysis tables were produced with flextable. Descriptive statistics were calculated for all relevant variables. Missing data were handled by listwise (complete-case) exclusion; lactate dehydrogenase and ejection fraction each had one missing observation, yielding *n* = 41 for these two variables in the corresponding univariable Cox models (Table 3); all other variables were complete for the full cohort (*n* = 42). Normality of numerical variables was assessed with the Shapiro–Wilk test. Parametric numerical data are reported as mean ± standard deviation (SD); non-parametric numerical data are reported as median (minimum–maximum). Categorical data are reported as *n* (%). Between-group comparisons of numerical variables used the independent-samples *t*-test (two groups) or ANOVA (>2 groups) for normally distributed data, and the Wilcoxon rank-sum test (two groups) or Kruskal–Wallis test (>2 groups) for non-normally distributed data. Categorical variables were compared using the Pearson chi-square test when expected cell counts were adequate (>5 per cell) and Fisher’s exact test otherwise. Survival analysis used the Kaplan–Meier method, with between-group differences assessed by the log-rank test. Univariable Cox proportional-hazards regression was used to estimate hazard ratios (HR) with 95% confidence intervals (CI) for OS and PFS; as detailed in Section 2.2, complete response and refractory disease were excluded from these models and are instead reported descriptively (Table 2 and Table 4). Given the modest sample size relative to the number of candidate biomarkers, univariable Cox regression for OS and PFS was restricted, among the inflammation-nutrition indices, to NLR, PNI, and NPAR; CAR and SII were instead compared descriptively, together with the Karnofsky Performance Score, between vital-status groups (Section 3.3, Table 5). A two-sided *p*-value < 0.05 was considered statistically significant throughout. Median follow-up was estimated using the reverse Kaplan–Meier method (median follow-up, 67 months; 95% CI, 37–132). Some subgroups—notably the lowest Charlson Comorbidity Index category (*n* = 1), the highest IPS risk group (*n* = 2), and rare treatment-regimen categories—contained very few patients, and some strata had zero or 100% events; hazard-ratio estimates for these strata are therefore unstable (in some cases producing extremely large point estimates with 0–∞ confidence intervals) and are reported descriptively rather than as reliable effect estimates (Section 3.3, Section 3.4 and Section 3.5 and Section 4.1).

## 3. Results

### 3.1. Baseline Characteristics

Forty-two patients aged ≥60 years with Hodgkin lymphoma were included: 41 with classical Hodgkin lymphoma and 1 with nodular lymphocyte-predominant Hodgkin lymphoma (Section 2.1). Median age at diagnosis was 68 years (range, 60–94), and 24 patients (57.1%) were male. Comorbidity burden was high: 30 patients (71.4%) had a high-risk (score 4–5) and 8 (19.0%) had a very high-risk (score 7–8) Charlson Comorbidity Index, while only 1 patient (2.4%) had a low-risk score. Functional status at diagnosis was frequently impaired, with 14 patients (33.3%) spending more than 50% of the day in bed (ECOG 3) and 16 (38.1%) classified as having a poor Karnofsky score (<50). Most patients (59.5%) fell into the intermediate IPS risk group (score 3–4). B symptoms were present in 40 patients (95.2%), and advanced-stage disease (III–IV) was present in 21 patients (50.0%). Splenic involvement was documented in 15 patients (35.7%) and extranodal involvement in 8 (19.0%). Full baseline demographic, clinical, and laboratory characteristics are shown in Table 1.

### 3.2. Treatment and Outcomes

Most patients (27, 64.3%) received ABVD as first-line therapy, and 6 (14.3%) received AVD (bleomycin-omitted); the remainder received alternative regimens including BV-AVD, R-CHOP, GDP, brentuximab vedotin/nivolumab-containing combinations, or no antineoplastic treatment (2 patients, 4.8%), as detailed in Table 2. A single line of treatment was sufficient in 34 patients (81.0%). Complete response was achieved in 28 patients (66.7%), while 6 patients (14.3%) had primary refractory disease; both variables are reported here descriptively only and were not included in the Cox or Kaplan–Meier prognostic models (Section 2.2). Bleomycin dose reduction or discontinuation was required in 3 patients (7.1%), and cardiac or pulmonary toxicity was each documented in 2 of 36 evaluable patients (5.6%). The PFS analysis comprised 21 events: 9 deaths without prior relapse or refractory disease, 5 deaths preceded by relapse, 4 deaths preceded by refractory disease, 2 deaths preceded by both relapse and refractory disease, and 1 additional event in a patient who relapsed twice but remained alive at last follow-up. The median observed time to death or last follow-up was 29.5 months (range, 1–159), and the median observed time to a PFS event or last follow-up was 21.5 months (range, 1–159); these are raw, unadjusted observed durations rather than Kaplan–Meier survival estimates, and are distinct from the Kaplan–Meier median OS (88 months) and median PFS (72 months) reported in the Abstract and derived in Section 3.3, Section 3.4 and Section 3.5, which account for censoring among patients still alive or event-free at last follow-up. At last follow-up, 20 patients (47.6%) had died and 22 (52.4%) remained alive. Full treatment and outcome characteristics are shown in Table 2.

### 3.3. Univariable Cox Regression for Overall Survival

On univariable Cox proportional-hazards regression, older age at diagnosis was associated with inferior OS (HR 1.09 per year, 95% CI 1.03–1.16, *p* = 0.002). Functional status was strongly prognostic: patients spending more than 50% of the day in bed (ECOG 3) had markedly higher mortality risk than those only mildly restricted (HR 22.4, 95% CI 2.87–174.8, *p* = 0.003). A high IPS (HR 11.1, 95% CI 1.87–66.1, *p* = 0.007) and stage IV disease (HR 6.24, 95% CI 1.12–34.70, *p* = 0.036) were both associated with worse OS. Splenic involvement (HR 4.33, 95% CI 1.72–10.90, *p* = 0.002) and extranodal involvement (HR 6.55, 95% CI 2.11–20.33, *p* = 0.001) were among the strongest adverse prognostic factors identified.

Among laboratory parameters, lower hemoglobin (HR 0.80 per g/dL, *p* = 0.024), lower albumin (HR 0.32 per g/dL, *p* = 0.004), and lower PNI score (HR 0.93 per unit, *p* = 0.006) were associated with inferior OS, while higher NLR (HR 1.07 per unit, *p* = 0.031) and higher NPAR (HR 1.08 per unit, *p* = 0.010) were associated with increased mortality risk; LDH reached statistical significance but with a negligible per-unit effect size (HR 1.00, *p* = 0.008). Regarding treatment, patients receiving AVD had a higher mortality risk than those receiving ABVD (HR 5.88, 95% CI 1.78–19.38, *p* = 0.004), and patients who received no antineoplastic treatment had the highest risk among treatment categories (HR 9.05, 95% CI 1.74–46.96, *p* = 0.009); these comparisons should be interpreted with caution given the small and clinically heterogeneous subgroups involved (see Section 3.5). Ejection fraction was not associated with OS (*p* = 0.199). As detailed in Section 2.2, complete response and refractory disease were not entered into this or any other Cox model in this study because of guarantee-time bias and, for refractory disease, circularity with the composite PFS endpoint; their unadjusted descriptive relationship to vital status is instead reported below and in Table 4. Full Cox regression results for OS are shown in Table 3.

Because CAR and SII were not included in the Cox regression models above, we present here a descriptive comparison of these indices, together with NPAR and the Karnofsky Performance Score, between patients who died (*n* = 20) and those who remained alive (*n* = 22) at last follow-up (Wilcoxon rank-sum test or Student’s *t*-test for continuous variables; Fisher’s exact test for the categorical Karnofsky score). NPAR was significantly higher in patients who died (24.89 ± 9.46) than in those who survived (20.16 ± 4.92; t = −2.06, df = 40, *p* = 0.046); this was the only statistically significant comparison among the inflammation-nutrition indices examined (Table 4). The Karnofsky Performance Score was significantly associated with vital status (Fisher’s exact test, *p* = 0.001): patients who died were classified with a poor score (<50) more frequently (13/20, 65.0%) than those who survived (3/22, 13.6%). CAR (median 27.88 [0.98–113.62] in patients who died vs. 6.61 [0.49–87.08] in survivors; *p* = 0.052) approached but did not reach statistical significance, and SII (729.54 [73.07–8639.42] vs. 986.33 [259.17–4672.76]; *p* = 0.141) did not differ significantly between vital-status groups.

### 3.4. Univariable Cox Regression for Progression-Free Survival

With PFS defined as the composite endpoint described in Section 2.2 (relapse, refractory disease, or death from any cause), the pattern of association with PFS closely paralleled that seen for OS. Older age (HR 1.07 per year, *p* = 0.005), ECOG performance status with more than 50% of the day spent in bed (HR 11.1, 95% CI 2.43–50.28, *p* = 0.002), high IPS (HR 17.7, 95% CI 2.69–116.5, *p* = 0.003), splenic involvement (HR 3.82, 95% CI 1.58–9.21, *p* = 0.003), and extranodal involvement (HR 4.32, 95% CI 1.51–12.32, *p* = 0.006) were all significantly associated with worse PFS; stage IV disease showed a similar but non-significant trend (HR 4.49, *p* = 0.077). Among laboratory parameters, hemoglobin (HR 0.78 per g/dL, *p* = 0.010), albumin (HR 0.43 per g/dL, *p* = 0.018), LDH (*p* = 0.005), and PNI score (HR 0.94 per unit, *p* = 0.027) were significantly associated with PFS; NPAR was also significantly associated with PFS (HR 1.07 per unit, *p* = 0.033), while NLR was not (*p* = 0.101). As detailed in Section 2.2, complete response and refractory disease were excluded from this Cox model because refractory disease is itself a component of the composite PFS endpoint, making its analysis as a PFS predictor circular, and complete response is subject to the same guarantee-time bias described for OS; both are reported descriptively only (Table 2). Several treatment-regimen categories (AVD, no treatment, and—less definitively—ABVD × 4 + radiotherapy + DHAP and ABVD × 4 + brentuximab vedotin + nivolumab) showed high point estimates for a PFS event, though these comparisons involve small, heterogeneous subgroups (Section 3.5 and Section 4.1). Full results are shown in Table 5.

### 3.5. Kaplan–Meier Analysis and Log-Rank Testing

Kaplan–Meier estimation with log-rank testing was performed for six clinically relevant grouping variables (Table 6); complete response and refractory disease were excluded from this analysis for the reasons given in Section 2.2. Sex was not associated with either OS or PFS (log-rank *p* = 0.373 and *p* = 0.463, respectively). ECOG performance status was strongly associated with both OS (χ^2^ = 24.109, *p* < 0.001) and PFS (χ^2^ = 18.591, *p* < 0.001). IPS risk group was significantly associated with both OS (χ^2^ = 9.774, *p* = 0.008) and PFS (χ^2^ = 13.552, *p* = 0.001). Ann Arbor stage (early [I–II] vs. advanced [III–IV]) was significantly associated with both OS (χ^2^ = 7.358, *p* = 0.007) and PFS (χ^2^ = 7.930, *p* = 0.005). Both splenic involvement (OS *p* = 0.001; PFS *p* = 0.001) and extranodal involvement (OS *p* < 0.001; PFS *p* = 0.002) were significantly associated with inferior survival on both endpoints. In contrast to a relapse-only PFS definition used in an earlier draft—under which ECOG status appeared unrelated to PFS (*p* = 0.542), suggesting a dissociation between performance status and progression risk—the composite PFS definition used here (Section 2.2) shows that ECOG performance status, IPS risk group, and Ann Arbor stage are each significantly associated with both survival endpoints, indicating that the previously apparent dissociation largely reflected the relapse-only PFS definition rather than a genuine biological finding (Section 4.1). Kaplan–Meier curves for the principal significant OS and PFS comparisons are shown in Figure 1 and Figure 2, respectively.

## 4. Discussion

In this retrospective cohort of 42 patients aged ≥60 years with Hodgkin lymphoma, we found a heavily comorbid population—over 90% with a moderate-to-very-high Charlson Comorbidity Index—in whom performance status, IPS risk group, extranodal and splenic disease involvement, and inflammatory-nutritional biomarkers were each associated with inferior survival. Complete response and refractory disease also showed a marked descriptive relationship with outcome. These findings are broadly consistent with, and extend, the growing body of literature describing the distinct clinical trajectory of Hodgkin lymphoma in older adults [2,4,5,7,12].

In this cohort, poor ECOG performance status was associated with a marked increase in both mortality risk (HR 22.4, *p* = 0.003) and PFS-event risk (HR 11.1, *p* = 0.002), emerging as a strong prognostic indicator in elderly Hodgkin lymphoma. Although the IPS is a well-established prognostic tool in advanced-stage Hodgkin lymphoma, the limited representation of older patients in its original development cohort, and the fact that the score does not capture frailty, comorbidity, or functional status, underscore the importance of supplementary prognostic assessment in this patient group [12]. Indeed, current data indicate that frailty and comorbidity measures provide prognostic information complementary to classic disease-based risk indicators in elderly Hodgkin lymphoma patients [14]. That the IPS continued to discriminate both OS and PFS in our study supports its prognostic value in older patients, while the strong association of ECOG performance status with both endpoints emphasizes that functional status, in addition to disease burden, should be incorporated into risk assessment. Nonetheless, given the single-center retrospective design and limited sample size, our findings require validation in larger, prospective, multicenter cohorts.

The magnitude of the association we observed between age and OS (HR 1.09 per year) is concordant with large population-based data: in a Norwegian case–control study of 492 patients aged ≥60 years, five-year OS ranged from as low as 9–20% among patients ineligible for or receiving only palliative treatment to 64% among those receiving curative-intent therapy, and treatment-related mortality even in curatively treated patients approached 6.5% [4]. Similarly, poor ECOG performance status was the strongest clinical predictor of both OS and PFS in our cohort (HR 22.4 and HR 11.1, respectively, for patients bed-bound >50% of the day), mirroring recent work showing that simplified frailty indices combining age, ECOG status, and a Cumulative Illness Rating Scale-based comorbidity score [13] stratify five-year OS from 86% (fit) to 22% (frail) and treatment-related mortality from 1% to 22% across frailty categories in older classical Hodgkin lymphoma [14]. Notably, ECOG status was prognostic for both OS and PFS in our cohort, indicating that poor performance status was associated with both overall mortality and disease progression/relapse risk in this cohort. Structured screening instruments such as the G8 geriatric screening tool, validated across solid and hematologic malignancies, offer a pragmatic, prospective means of capturing frailty alongside ECOG and may help refine risk stratification and treatment-intensity decisions in future cohorts of elderly Hodgkin lymphoma patients, usefully complementing ECOG assessment [22].

Our data support describing this cohort as heavily comorbid—over 90% of patients fell into the moderate-to-very-high Charlson Comorbidity Index categories—but they do not, on their own, demonstrate a statistically robust CCI–survival association. Comorbidity burden has been reported elsewhere as a powerful discriminator of outcome, in some series more so than chronological age alone: in a Hungarian cohort of 35 patients aged ≥60 years, a Charlson Comorbidity Index >7 was associated with a five-year PFS of only 41.3% compared with 91.3% in patients with a lower score, whereas outcomes did not differ significantly across age subgroups [7]. In our own cohort, however, the univariable Cox estimates for CCI categories were numerically unstable, with extremely wide, non-informative confidence intervals driven by sparse strata and near-complete separation (very few or zero events in some categories, most notably the single-patient low-risk stratum; Table 3, Table 5); these estimates should not be read as evidence for or against a CCI–survival relationship in this dataset. What our data do support is that comorbidity assessment, not chronological age cutoffs alone, should inform risk stratification and treatment-intensity decisions in this population, consistent with current recommendations to incorporate geriatric and comorbidity assessment into first-line treatment planning for elderly Hodgkin lymphoma patients [12]; a definitive test of the CCI–survival relationship in our own cohort will require a larger, adequately powered sample.

Although the IPS was derived predominantly from a younger population with advanced-stage disease [1], it remained prognostic for both OS and PFS in our elderly cohort, supporting its continued relevance as a baseline biological risk classifier even though it does not by itself capture comorbidity or functional reserve. Splenic and extranodal involvement—markers of greater disease burden and dissemination—were among the strongest adverse prognostic factors for both OS and PFS in our analysis, consistent with their more generally established role as adverse prognostic features in Hodgkin lymphoma, including the higher rates of extranodal and unfavorable-histology disease reported in Epstein–Barr virus-associated classical Hodgkin lymphoma in older populations [10]. Likewise, the inflammatory and nutritional biomarkers evaluated (hemoglobin, albumin, PNI, and NLR) were each significantly associated with OS, consistent with a growing literature linking host inflammatory and nutritional status at diagnosis to survival in classical Hodgkin lymphoma—including a large multicenter analysis of nearly 1000 patients in which elevated NLR independently predicted inferior outcome [15]—and with the broader oncology literature from which the PNI [16] and systemic immune-inflammation index [21] were originally derived and subsequently adapted to lymphoid malignancies. These readily available, low-cost hematologic indices may usefully complement IPS and comorbidity scores in elderly patients, for whom more resource-intensive geriatric assessment tools are not always feasible in routine practice. In our cohort, the neutrophil percentage-to-albumin ratio (NPAR) was significantly higher in patients who died at last follow-up than in survivors (24.89 ± 9.46 vs. 20.16 ± 4.92; *p* = 0.046), a finding consistent with current literature linking NPAR, as a marker of systemic inflammation and nutritional deficiency, to mortality across various malignancy and critical-illness populations [20,23]. Although NPAR does not yet have a classical Hodgkin lymphoma-specific prognostic validation, the demonstrated integration of PNI and the leukocyte-lymphocyte ratio into the IPS [16] and the established prognostic significance of SII in diffuse large B-cell lymphoma [21] support the notion that inflammation-nutrition-based indices may carry complementary prognostic value in lymphoid malignancies. NPAR may therefore be considered a low-cost, easily calculable candidate biomarker warranting further validation studies.

Complete response and refractory disease showed a marked descriptive association with OS and PFS in this cohort (Table 2), with longer observed survival among patients who achieved complete response and shorter observed survival among those with refractory disease. However, because treatment response is a time-dependent variable determined after treatment, and refractory disease is itself a component of the PFS event definition, we deliberately excluded both variables from the Cox and Kaplan–Meier prognostic models (Section 2.2) rather than reporting them as independent prognostic effects. The present results therefore do not permit a direct inference regarding treatment intensity, but they do support the importance of basing treatment decisions in elderly Hodgkin lymphoma patients not on chronological age alone, but on an integrated assessment of performance status, comorbidities, and treatment tolerance. The current approach favors adapting treatment to patient fitness and early treatment response rather than reducing treatment intensity on the basis of age alone. Interim PET-CT-guided treatment adaptation has been shown to allow safe de-escalation in patients with a good response in both early-stage [24] and advanced-stage [25]. Hodgkin lymphoma, while guiding treatment intensification in patients with an inadequate early response. De-escalation studies in early-stage disease similarly show that treatment intensity, including bleomycin exposure, can be reduced in selected patients without compromising disease control [26]. In our cohort, most patients received standard-dose ABVD despite a high comorbidity burden, and bleomycin dose reduction or discontinuation was required in only a small number of patients. Nonetheless, a pooled GHSG analysis of older early-stage patients reported that omitting bleomycin from treatment can reduce pulmonary toxicity while preserving efficacy in selected patients [9]. Alternative treatment options are particularly important in patients for whom bleomycin use is not appropriate or standard chemotherapy tolerance is limited. Frontline brentuximab vedotin–AVD has been compared with ABVD in randomized trials and shown to be an effective option in advanced-stage disease [27]; however, the populations of these trials have limited representation of frail elderly patients. Combinations of brentuximab vedotin with dacarbazine or with a PD-1 inhibitor such as nivolumab have provided high response rates and manageable toxicity profiles, particularly in elderly patients not eligible for chemotherapy [5,28]. Our observations regarding the small number of patients in our cohort treated with brentuximab vedotin- or immune checkpoint inhibitor-containing regimens are consistent with the feasibility of these approaches; however, comparative efficacy assessment across regimens could not be performed because of the small subgroup sizes.

### 4.1. Limitations

This study has several limitations that warrant consideration. First, it is a retrospective, single-center analysis with a modest sample size (*n* = 42), which limits statistical power for both endpoints, particularly given that several covariates (the Charlson Comorbidity Index, IPS-5, and some treatment-regimen categories) contained very few patients in one or more strata (Table 2, Section 4.1). Second, several subgroup categories within the Charlson Comorbidity Index and some treatment-regimen strata contained very few or zero events, producing extremely large and unstable hazard ratios with non-informative (0–∞) confidence intervals; these estimates reflect statistical instability rather than genuine effect sizes and must be interpreted with caution—in particular, our data should not be read as demonstrating a robust CCI–survival association in this cohort (Section 4). Third, only univariable Cox regression was performed; given the limited number of events, a multivariable model simultaneously adjusting for age, comorbidity, performance status, IPS, disease burden, and treatment-response variables was not feasible without a substantial risk of overfitting, and the reported associations should therefore not be interpreted as independent of one another. Fourth, treatment regimens were heterogeneous and were not randomly allocated, so regimen-outcome comparisons are subject to confounding by indication (e.g., preferential allocation of frailer or higher-risk patients to non-ABVD regimens). Fifth, complete response and refractory disease, although strongly associated with outcome descriptively, were excluded from all Cox and Kaplan–Meier prognostic models because they are post-treatment, time-dependent variables subject to guarantee-time bias, and because refractory disease is itself a component of the composite PFS endpoint, making its use as a PFS predictor circular; landmark or time-dependent Cox modeling, which could in principle address this, was not feasible given the limited number of events in this cohort, and this should be pursued in future, larger studies. Sixth, some variables (including viral serology markers) had missing data, and the single-center design may limit generalizability to other populations and treatment settings. Seventh, no standardized geriatric screening instrument (e.g., the G8 tool) was applied at diagnosis [22], so the relative contribution of ECOG performance status, versus broader geriatric domains (cognition, mobility, nutrition, polypharmacy), to overall and progression-free survival could not be disentangled. Eighth, the cohort included one case of nodular lymphocyte-predominant Hodgkin lymphoma (NLPHL) alongside 41 classical Hodgkin lymphoma cases; because NLPHL is a biologically and clinically distinct entity for which the IPS was not developed, this introduces a limited degree of histologic heterogeneity, and results should be interpreted with this in mind (Section 2.1). Finally, as with any retrospective cohort, unmeasured confounding and information bias inherent to medical-record data cannot be excluded.

### 4.2. Conclusions

In this real-world cohort of elderly patients with Hodgkin lymphoma, poor performance status, high IPS risk group, and splenic and extranodal disease involvement, together with adverse inflammatory-nutritional biomarkers (low hemoglobin, low albumin, low PNI, and high NLR), were each associated with inferior survival. The cohort was also heavily comorbid, though our data support describing this burden rather than claiming a robust CCI–survival association given the sparse strata and unstable hazard-ratio estimates involved. Complete response and refractory disease showed a strong descriptive association with outcome (Table 2; Section 2.2) but were intentionally excluded from the prognostic Cox/Kaplan–Meier models because of their post-treatment, time-dependent nature and, for refractory disease, circularity with the PFS endpoint definition. These findings support a management approach that integrates conventional IPS-based staging with geriatric, comorbidity, and biomarker-based risk assessment, prioritizes strategies that maximize the likelihood of complete response, and considers less toxic or novel-agent-based regimens for patients less able to tolerate standard ABVD. Larger, prospective, multicenter studies with sufficient events—ideally incorporating multivariable and landmark/time-dependent modeling—are needed to validate these findings and to develop elderly-specific prognostic models for Hodgkin lymphoma.

## Figures and Tables

**Table 1:** Demographic, clinical, and laboratory characteristics of the study population (*n* = 42).

Characteristic	Value
Age at diagnosis, years, median (range)	68 (60–94)
Sex, *n* (%)	
Male	24 (57.1)
Female	18 (42.9)
Disease duration before diagnosis, months, median (range)	6 (1–44)
Charlson Comorbidity Index, *n* (%)	
0–1 (low risk)	1 (2.4)
2–3 (moderate risk)	3 (7.1)
4–5 (high risk)	30 (71.4)
7–8 (very high risk)	8 (19.0)
Karnofsky Performance Score, *n* (%)	
Good (≥80)	8 (19.0)
Moderate (50–70)	18 (42.9)
Poor (<50)	16 (38.1)
ECOG performance status, *n* (%)	
Mildly restricted (ECOG 1)	13 (31.0)
Bed-bound <50% of day (ECOG 2)	15 (35.7)
Bed-bound >50% of day (ECOG 3)	14 (33.3)
International Prognostic Score (IPS) group, *n* (%)	
1–2 (low)	15 (35.7)
3–4 (intermediate)	25 (59.5)
5 (high)	2 (4.8)
Histologic subtype, *n* (%)	
Nodular sclerosis	10 (23.8)
Mixed cellularity	12 (28.6)
Lymphocyte-depleted	10 (23.8)
Lymphocyte-rich	9 (21.4)
Lymphocyte-predominant (NLPHL) †	1 (2.4)
Ann Arbor stage, *n* (%)	
I	5 (11.9)
II	16 (38.1)
III	12 (28.6)
IV	9 (21.4)
B symptoms, *n* (%)	
Absent	2 (4.8)
Present	40 (95.2)
Splenic involvement, *n* (%)	
Absent	27 (64.3)
Present	15 (35.7)
Extranodal involvement, *n* (%)	
Absent	34 (81.0)
Present	8 (19.0)
Laboratory parameters, mean ± SD or median (range)	
Hemoglobin, g/dL	11.83 ± 2.19
White blood cell count, ×10^3^/µL	7.93 (2.97–25.32)
Lymphocyte count, ×10^3^/µL	1.47 (0.31–6.20)
Neutrophil count, ×10^3^/µL	5.29 (0.53–24.11)
Neutrophil percentage, %	67.47 ± 13.80
Platelet count, ×10^3^/µL	245.5 (24–564)
Erythrocyte sedimentation rate, mm/h	37.79 ± 22.26
Creatinine, mg/dL	0.80 (0.49–1.74)
C-reactive protein, mg/L	52.85 (1.8–342)
Albumin, g/dL	3.20 ± 0.68
Lactate dehydrogenase, U/L	250 (139–807)
Uric acid, mg/dL	4.2 (1.22–11.3)
Ferritin, ng/mL	273 (25.4–2669)
Neutrophil-to-lymphocyte ratio (NLR)	3.60 (0.31–40.18)
CRP-to-albumin ratio (CAR)	18.07 (0.49–113.62)
Neutrophil percentage-to-albumin ratio (NPAR)	22.41 ± 7.72
Systemic immune-inflammation index (SII)	955.65 (73.07–8639.42)
Prognostic Nutritional Index (PNI) score	40.49 ± 9.64

† This single case of nodular lymphocyte-predominant Hodgkin lymphoma (NLPHL) is biologically and clinically distinct from the 41 classical Hodgkin lymphoma cases above and is listed separately rather than as a classical-subtype category (Section 2.1).

**Table 3:** Univariable Cox proportional-hazards regression for overall survival.

Characteristic	*n*	HR	95% CI	*p*
Age at diagnosis (per year)	42	1.09	1.03–1.16	0.002
Sex	42			
Male		—	—	
Female		0.63	0.24–1.65	0.351
Charlson Comorbidity Index	42			
0–1 (low risk)		—	—	
2–3 (moderate risk)		2.20 × 10^9^	0–∞	0.999
4–6 (high risk)		6.85 × 10^9^	0–∞	0.998
7–8 (very high risk)		1.60 × 10^10^	0–∞	0.998
ECOG performance status	42			
Mildly restricted		—	—	
Bed-bound <50% of day		2.31	0.49–10.93	0.291
Bed-bound >50% of day		22.4	2.87–174.8	0.003
IPS risk score	42			
1–2 (low)		—	—	
3–4 (intermediate)		1.79	0.68–4.72	0.239
5 (high)		11.1	1.87–66.1	0.007
Ann Arbor stage	42			
I		—	—	
II		1.13	0.23–5.62	0.882
III		2.47	0.49–12.55	0.275
IV		6.24	1.12–34.70	0.036
B symptoms (present vs. absent)	42	2.20 × 10^10^	0–∞	0.999
Splenic involvement (present vs. absent)	42	4.33	1.72–10.90	0.002
Extranodal involvement (present vs. absent)	42	6.55	2.11–20.33	0.001
Hemoglobin (per g/dL)	42	0.80	0.66–0.97	0.024
Albumin (per g/dL)	42	0.32	0.15–0.70	0.004
LDH (per U/L)	41	1.00	1.00–1.01	0.008
NLR (per unit)	42	1.07	1.01–1.14	0.031
PNI score (per unit)	42	0.93	0.88–0.98	0.006
NPAR (per unit)	42	1.08	1.02–1.15	0.010
Treatment regimen	42			
ABVD		—	—	
AVD		5.88	1.78–19.38	0.004
BV-AVD		0.00	0–∞	0.999
R-CHOP		0.00	0–∞	0.999
GDP		0.00	0–∞	0.998
No treatment		9.05	1.74–46.96	0.009
ABVD × 4 + RT + DHAP		1.67	0.21–13.49	0.629
ABVD × 4 + brentuximab + nivolumab		3.23	0.39–26.48	0.276
ABVD × 2 + BV × 4 + dacarbazine		0.00	0–∞	0.999
Ejection fraction (per %)	41	0.97	0.91–1.02	0.199

Abbreviations: CI, confidence interval; HR, hazard ratio; IPS, International Prognostic Score; LDH, lactate dehydrogenase; NLR, neutrophil-to-lymphocyte ratio; NPAR, neutrophil percentage-to-albumin ratio; PNI, Prognostic Nutritional Index; RT, radiotherapy. Reference categories are denoted with “—”. Extremely large HRs with 0–∞ confidence intervals (e.g., within the Charlson Comorbidity Index and some treatment-regimen subgroups) reflect near-complete separation from very low or zero event counts in those subgroups and denote statistical instability rather than a reliable effect size; these should not be interpreted as genuine effect sizes. Complete response and refractory disease are not included in this model (Section 2.2; see Table 4 for their descriptive relationship to vital status).

**Table 4:** Descriptive comparison of CAR, NPAR, SII, and Karnofsky Performance Score by vital status.

Variable	Deceased (*n* = 20)	Alive (*n* = 22)	*p* Value
CRP-to-albumin ratio (CAR), median (range)	27.88 (0.98–113.62)	6.61 (0.49–87.08)	0.052
Neutrophil percentage-to-albumin ratio (NPAR), mean ± SD	24.89 ± 9.46	20.16 ± 4.92	0.046
Systemic immune-inflammation index (SII), median (range)	729.54 (73.07–8639.42)	986.33 (259.17–4672.76)	0.141
Poor Karnofsky Performance Score (<50), *n* (%)	13/20 (65.0)	3/22 (13.6)	0.001

Abbreviations: CAR, C-reactive protein-to-albumin ratio; NPAR, neutrophil percentage-to-albumin ratio; SII, systemic immune-inflammation index. The Wilcoxon rank-sum test or Student’s *t*-test was used for continuous variables, and Fisher’s exact test for the categorical Karnofsky score. CAR and SII were not included in the Cox regression models above and are reported descriptively here; NPAR was also included in the Cox models in Table 3 and Table 5. Significant *p*-values are bolded.

**Table 5:** Univariable Cox proportional-hazards regression for progression-free survival.

Characteristic	*n*	HR	95% CI	*p*
Age at diagnosis (per year)	42	1.07	1.02–1.13	0.005
Sex	42			
Male		—	—	
Female		0.73	0.30–1.75	0.475
Charlson Comorbidity Index	42			
0–1 (low risk)		—	—	
2–3 (moderate risk)		1.24 × 10^9^	0–∞	0.998
4–6 (high risk)		2.82 × 10^9^	0–∞	0.998
7–8 (very high risk)		6.81 × 10^9^	0–∞	0.998
ECOG performance status	42			
Mildly restricted		—	—	
Bed-bound <50% of day		3.01	0.62–14.53	0.170
Bed-bound >50% of day		11.1	2.43–50.28	0.002
IPS risk score	42			
1–2 (low)		—	—	
3–4 (intermediate)		2.35	0.84–6.56	0.103
5 (high)		17.7	2.69–116.5	0.003
Ann Arbor stage	42			
I		—	—	
II		0.87	0.16–4.57	0.868
III		2.73	0.56–13.24	0.213
IV		4.49	0.85–23.72	0.077
B symptoms (present vs. absent)	42	2.74 × 10^10^	0–∞	0.999
Splenic involvement (present vs. absent)	42	3.82	1.58–9.21	0.003
Extranodal involvement (present vs. absent)	42	4.32	1.51–12.32	0.006
Hemoglobin (per g/dL)	42	0.78	0.65–0.94	0.010
Albumin (per g/dL)	42	0.43	0.21–0.86	0.018
LDH (per U/L)	41	1.00	1.00–1.01	0.005
NLR (per unit)	42	1.05	0.99–1.12	0.101
PNI score (per unit)	42	0.94	0.90–0.99	0.027
NPAR (per unit)	42	1.07	1.01–1.14	0.033
Treatment regimen	42			
ABVD		—	—	
AVD		5.65	1.77–18.00	0.003
BV-AVD		7.36	0.84–64.25	0.071
R-CHOP		0.00	0–∞	0.982
GDP		0.00	0–∞	1.000
No treatment		26.4	4.18–167.3	0.001
ABVD × 4 + RT + DHAP		5.53	0.65–47.07	0.117
ABVD × 4 + brentuximab + nivolumab		5.53	0.65–47.07	0.117
ABVD × 2 + BV × 4 + dacarbazine		0.00	0–∞	1.000
Ejection fraction (per %)	41	0.96	0.91–1.00	0.073

Abbreviations as in Table 3. Complete response and refractory disease are not included in this model because refractory disease is a component of the composite PFS endpoint itself (Section 2.2); see Table 2 for their descriptive frequencies.

**Table 6:** Log-rank test results for Kaplan–Meier analysis of overall survival and progression-free survival by grouping variable.

Grouping Variable	Overall Survival			Progression-Free Survival		
	χ^2^	df	*p*	χ^2^	df	*p*
Sex	0.794	1	0.373	0.540	1	0.463
ECOG performance status	24.109	2	<0.001	18.591	2	<0.001
IPS risk group	9.774	2	0.008	13.552	2	0.001
Ann Arbor stage (I–II vs. III–IV)	7.358	1	0.007	7.930	1	0.005
Splenic involvement	11.733	1	0.001	10.679	1	0.001
Extranodal involvement	14.332	1	<0.001	9.467	1	0.002

Complete response and refractory disease were excluded from Kaplan–Meier/log-rank analysis because of the guarantee-time bias and endpoint-circularity concerns described in Section 2.2; their descriptive frequencies are reported in Table 2. Significant *p*-values are bolded in the original data output.

## Data Availability

The data presented in this study are available from the corresponding author upon reasonable request. The data are not publicly available because they contain information that could compromise patient privacy and confidentiality.

## References

[1] Hasenclever D., Diehl V. (1998). International Prognostic Factors Project on Advanced Hodgkin’s Disease. A prognostic score for advanced Hodgkin’s disease. N. Engl. J. Med..

[2] Ansell S.M. (2025). Hodgkin lymphoma: 2025 update on diagnosis, risk-stratification, and management. Am. J. Hematol..

[3] Canellos G.P., Anderson J.R., Propert K.J., Nissen N., Cooper M.R., Henderson E.S., Green M.R., Gottlieb A., Peterson B.A. (1992). Chemotherapy of advanced Hodgkin’s disease with MOPP, ABVD, or MOPP alternating with ABVD. N. Engl. J. Med..

[4] Lia K., Jørgensen R.R.K., LWold B., Fluge Ø., Fagerli U.-M., Bersvendsen H., Bø I.B., Bhargava S., Fosså A. (2024). Overall survival and causes of death in elderly patients with Hodgkin lymphoma: A Norwegian population-based case-control study. Haematologica.

[5] Friedberg J.W., Bordoni R., Patel-Donnelly D., Larson T., Goldschmidt J., Boccia R., Cline V.J., Mamidipalli A., Liu J., Akyol A. (2024). Brentuximab vedotin with dacarbazine or nivolumab as frontline classical Hodgkin lymphoma therapy for older patients ineligible for chemotherapy. Blood.

[6] Charlson M.E., Pompei P., Ales K.L., MacKenzie C.R. (1987). A new method of classifying prognostic comorbidity in longitudinal studies: Development and validation. J. Chronic Dis..

[7] Tóthfalusi D., Dobó B., Borics F., Pinczés L.I., Illés Á., Miltényi Z. (2025). Impact of comorbidities on prognosis and treatment outcomes in elderly patients with Hodgkin lymphoma. Clin. Pract..

[8] Diehl V., Franklin J., Pfreundschuh M., Lathan B., Paulus U., Hasenclever D., Tesch H., Herrmann R., Dörken B., Müller-Hermelink H.-K. (2003). Standard and increased-dose BEACOPP chemotherapy compared with COPP-ABVD for advanced Hodgkin’s disease. N. Engl. J. Med..

[9] German Hodgkin Study Group (2016). Bleomycin in older early-stage favorable Hodgkin lymphoma patients: Analysis of the German Hodgkin Study Group (GHSG) HD10 and HD13 trials. Blood.

[10] Jarrett R.F., Stark G.L., White J., Angus B., Alexander F.E., Krajewski A.S., Freeland J., Taylor G.M., Taylor P.R.A., Scotland and Newcastle Epidemiology of Hodgkin Disease Study Group (2005). Impact of tumor Epstein-Barr virus status on presenting features and outcome in age-defined subgroups of patients with classic Hodgkin lymphoma: A population-based study. Blood.

[11] Oken M.M., Creech R.H., Tormey D.C., Horton J., Davis T.E., McFadden E.T., Carbone P.P. (1982). Toxicity and response criteria of the Eastern Cooperative Oncology Group. Am. J. Clin. Oncol..

[12] Evens A.M., McKenna M., Ryu Tiger Y.K., Upshaw J.N. (2023). Hodgkin lymphoma treatment for older persons in the modern era. Hematol. Am. Soc. Hematol. Educ. Program.

[13] Miller M.D., Paradis C.F., Houck P.R., Mazumdar S., Stack J.A., Rifai A., Mulsant B., Reynolds C.F. (1992). Rating chronic medical illness burden in geropsychiatric practice and research: Application of the Cumulative Illness Rating Scale. Psychiatry Res..

[14] Lia K., Jørgensen R.R.K., Wikman P., Wold B.L., Övergaard N., Fluge Ø., Fagerli U.-M., Bersvendsen H., Bø I.B., Bhargava S. (2025). A simplified frailty score predicts outcome in curatively treated older patients with classical Hodgkin lymphoma. Haematologica.

[15] Marcheselli R., Bari A., Tadmor T., Marcheselli L., Cox M.C., Pozzi S., Ferrari A., Baldini L., Gobbi P., Aviv A. (2017). Neutrophil-lymphocyte ratio at diagnosis is an independent prognostic factor in patients with nodular sclerosis Hodgkin lymphoma: Results of a large multicenter study involving 990 patients. Hematol. Oncol..

[16] Paydas S., Lacin S., Dogan M., Barista I., Yildiz B., Seydaoglu G., Karadurmus N., Civriz S., Kaplan M.A., Yagci M. (2021). Easier and more explanatory indices by integrating leukocyte lymphocyte ratio (LLR) and prognostic nutritional index (PNI) to IPS systems in cases with classical Hodgkin lymphoma. Leuk. Res..

[17] Karnofsky D.A., Burchenal J.H., MacLeod C.M. (1949). The Clinical Evaluation of Chemotherapeutic Agents in Cancer. Evaluation of Chemotherapeutic Agents.

[18] Cheson B.D., Fisher R.I., Barrington S.F., Cavalli F., Schwartz L.H., Zucca E., Lister T.A. (2014). Recommendations for initial evaluation, staging, and response assessment of Hodgkin and non-Hodgkin lymphoma: The Lugano classification. J. Clin. Oncol..

[19] Swerdlow S.H., Campo E., Pileri S.A., Harris N.L., Stein H., Siebert R., Advani R., Ghielmini M., Salles G.A., Zelenetz A.D. (2016). The 2016 revision of the World Health Organization classification of lymphoid neoplasms. Blood.

[20] Hu C., He Y., Li J., Zhang C., Hu Q., Li W., Hao C. (2023). Association between neutrophil percentage-to-albumin ratio and 28-day mortality in Chinese patients with sepsis. J. Int. Med. Res..

[21] Wang Z., Zhang J., Luo S., Zhao X. (2021). Prognostic significance of systemic immune-inflammation index in patients with diffuse large B-cell lymphoma. Front. Oncol..

[22] Bellera C.A., Rainfray M., Mathoulin-Pélissier S., Mertens C., Delva F., Fonck M., Soubeyran P.L. (2012). Screening older cancer patients: First evaluation of the G-8 geriatric screening tool. Ann. Oncol..

[23] Li X., Wu M., Chen M., Liu R., Tao Q., Hu Y., Yu J., Chen D. (2025). The Association Between Neutrophil-Percentage-to-Albumin Ratio (NPAR) and Mortality Among Individuals With Cancer: Insights From National Health and Nutrition Examination Survey. Cancer Med..

[24] Radford J., Illidge T., Counsell N., Hancock B., Pettengell R., Johnson P., Wimperis J., Culligan D., Popova B., Smith P. (2015). Results of a trial of PET-directed therapy for early-stage Hodgkin’s lymphoma. N. Engl. J. Med..

[25] Johnson P., Federico M., Kirkwood A., Fosså A., Berkahn L., Carella A., D’amore F., Enblad G., Franceschetto A., Fulham M. (2016). Adapted treatment guided by interim PET-CT scan in advanced Hodgkin’s lymphoma. N. Engl. J. Med..

[26] Engert A., Plütschow A., Eich H.T., Lohri A., Dörken B., Borchmann P., Berger B., Greil R., Willborn K.C., Wilhelm M. (2010). Reduced treatment intensity in patients with early-stage Hodgkin’s lymphoma. N. Engl. J. Med..

[27] Connors J.M., Jurczak W., Straus D.J., Ansell S.M., Kim W.S., Gallamini A., Younes A., Alekseev S., Illés Á., Picardi M. (2018). Brentuximab vedotin with chemotherapy for stage III or IV Hodgkin’s lymphoma. N. Engl. J. Med..

[28] Ansell S.M., Lesokhin A.M., Borrello I., Halwani A., Scott E.C., Gutierrez M., Schuster S.J., Millenson M.M., Cattry D., Freeman G.J. (2015). PD-1 blockade with nivolumab in relapsed or refractory Hodgkin’s lymphoma. N. Engl. J. Med..

